# Synthesis of Few-Layer Graphene from Lignin and Its Application for the Creation of Thermally Conductive and UV-Protective Coatings

**DOI:** 10.3390/ma18235429

**Published:** 2025-12-02

**Authors:** Aleksei Vozniakovskii, Alexander Voznyakovskii, Anna Neverovskaya, Nikita Podlozhnyuk, Sergey Kidalov, Evgeny Auchynnikau

**Affiliations:** 1 Ioffe Institute, 194021 Saint Petersburg, Russia; 2 Institute for Synthetic Rubber, 198035 Saint Petersburg, Russia; 3 Department of Logistics and Management Methods, Yanka Kupala State University of Grodno, 230023 Grodno, Belarus

**Keywords:** lignin, waste, recycling, few-layer graphene, coating, self-propagating high-temperature synthesis, chemical cross-linking, thermal conductivity, UV protection

## Abstract

Coatings based on graphene nanostructures exhibit high thermal conductivity and are capable of effectively protecting materials from the negative effects of ultraviolet radiation. However, due to the imperfections of the methods for synthesizing graphene nanostructures and coatings based on them, the practical application of such coatings remains unprofitable. This paper presents the results of a study of the thermal conductivity and UV-protective properties of coatings synthesized by chemically crosslinking few-layer graphene particles on ABS plastic substrates. Few-layer graphene particles synthesized under self-propagating high-temperature synthesis conditions were used as the starting material for the coating synthesis. The synthesized coatings were found to have a thermal conductivity of 244 W/(m × K) and are capable of effectively protecting ABS plastic substrates from the negative effects of UV radiation, allowing the products to maintain their required strength characteristics. The high productivity of the method for synthesizing few-layer graphene (up to 10 kg/month at the laboratory production level), as well as the simplicity of the method for synthesizing coatings based on it, allows us to hope for the cost-effectiveness of such coatings.

## 1. Introduction

Industrial development inevitably leads to the generation of various wastes. One such waste is hydrolysis lignin. Hydrolysis lignin is an aromatic polymer with a complex structure and is a by-product of alcohol production from wood via the hydrolysis method [1]. Lignin accounts for 18–25% of wood volume and, in its dry form, is a dark brown powder that is insoluble in water [2]. Natural lignin is a complex organic molecule composed of three monomers: H-lignin (ρ-coumaryl alcohol), G-lignin (coniferyl alcohol), and S-lignin (sinapyl alcohol) units [3]. It should be noted that the ratio of these monomers can vary significantly depending on the specific type of plant (hardwood/softwood, etc.) [4], which greatly complicates their processing and application [5]. Over 100 million tons have accumulated in waste dumps worldwide, and an additional approximately 70 million tons of technical lignins are generated annually, of which no more than 2% is processed [6]. Lignin not only occupies vast territories but is also prone to forming slow-burning fires that are difficult to extinguish. Despite numerous attempts to use lignin in various sectors—from sorption materials and medical applications to additives in construction materials [7,8,9]—lignin has not yet found widespread application, and its processing remains an unsolved problem. Therefore, researchers are seeking new ways to process lignin into a useful product.

Few-layer graphene (FLG) is a type of graphene nanostructure (GNS) consisting of 3 to 10 graphene layers [10], which allows it to partially retain the characteristic properties of single-layer graphene. It should be noted that ideal single-layer graphene has a thermal conductivity of ~5000 W/(m·K) [11], while its Young’s modulus is 1 TPa, and its specific surface area is estimated at 2630 m^2^/g [12].

Due to their characteristics, FLG is a promising material for creating coatings for various purposes: corrosion protection [13], UV protection [14], antimicrobial coatings [15], thermally conductive [16], and electrically conductive coatings [17].

Furthermore, GNSs, and FLG in particular, are actively used by researchers as a modifying additive in the creation of polymer composite coatings with high anti-corrosion [18,19,20], tribological [21], and thermophysical [22] properties.

However, despite numerous publications where researchers have demonstrated successful testing of FLG-based coatings in various industries, these coatings are still not used in practice. The reason for this is the high production cost of all types of graphene nanostructures (GNSs), including few-layer graphene (FLG), which makes their application unprofitable. In turn, the high production cost of GNS, and FLG in particular, is due to the imperfection of GNS synthesis methods. Currently, all existing synthesis methods are divided into two main groups: “top-down” [23] and “bottom-up” [24] methods.

The “top-down” methods are based on the exfoliation of GNS from graphite. This group includes methods such as the Hummers method and its modifications, the ultrasonic exfoliation method using surfactants, and the mechanical exfoliation method. The advantage of these methods is their relatively high yield. However, these methods do not allow for the production of GNS with a low defect density.

The “bottom-up” methods are based on the synthesis of GNS from carbon-containing substances. This group of methods includes the Chemical Vapor Deposition (CVD) method, graphene synthesis on a SiC substrate, and others. These methods enable the production of high-quality GNS with a low defect density; however, their yield is extremely low.

Consequently, there is currently no synthesis methodology for FLG that allows for the production of large volumes of high-quality material at an acceptable cost.

One such method is self-propagating high-temperature synthesis (SHS). SHS is an exothermic chemical process of the combustion type, which proceeds in a self-propagating wave mode in powder mixtures and leads to the formation of useful condensed materials. SHS represents a mode of exothermic reaction in which heat release is localized in a narrow layer and is transferred from layer to layer by heat conduction [25]. For a long time, SHS was used for the synthesis of refractory materials: nitrides, carbides, and borides [26]. In our previous works [27], we demonstrated the possibility of synthesizing few-layer graphene (no more than 5 layers) from cyclic biopolymers, including lignin [28], under conditions of SHS. Unlike other methods, the proposed technology allows for the synthesis of large volumes of material, as well as the production of FLG (few-layer graphene) that does not contain Stone-Wales defects in its structure [29]. Due to this feature, the synthesized FLG demonstrated high efficiency in creating polymer composites based on photopolymer resins using DLP 3D printing [30].

The aim of this work was to establish the possibility of creating coatings from FLG particles that were synthesized from lignin under SHS process conditions, as well as to study their thermal conductivity and effectiveness in protecting polymer products from the negative effects of UV radiation.

## 2. Materials and Methods

### 2.1. Initial Material for Synthesis of Coatings

The source material for the synthesis of coatings was FLG (no more than 5 layers), synthesized under the conditions of the SHS process from hydrolytic lignin (waste dump of the Arkhangelsk pulp and paper mill, Russia). To synthesize the few-layer graphene, lignin powder was mixed with ammonium nitrate in a 1:1 ratio in a tumbler mixer. The resulting mixture was then placed in a reactor and heated to a temperature of 250 °C (the initiation temperature of the SHS process). The start of the SHS process was detected by the onset of active gas evolution, and the end of the SHS process was detected by the cessation of active gas evolution. After the synthesis was completed, the few-layer graphene powder was washed with water and dried in a drying oven at 110 °C until the mass loss ceased. The synthesis procedure is described in detail in [28]. Using the chemical method for quantitative detection of Stone-Wales defects [29], it was established that the synthesized few-layer graphene does not contain Stone-Wales defects in its structure within the sensitivity limits of the method.

### 2.2. Coating Synthesis Methodology

Graphene nanosheet edges have various surface groups [31,32]. Some of the most common types of surface groups are -OH and -COOH groups. In our previous works [16], it was established that such groups are also present in few-layer graphene. The presence of -OH groups allows us to perform chemical cross-linking of the few-layer graphene via these types of groups using the Zugeev–Tseretitinov reaction [33]. This reaction was carried out using diisoamyl ether and a Grignard reagent, both of analytical grade and supplied by Lenreaktiv, Russia.

**Figure 1:**
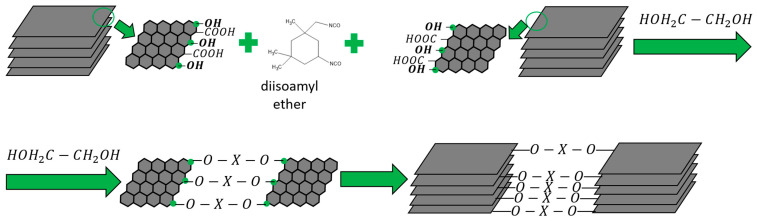
Synthesis scheme of coatings by chemical cross-linking of FLG particles.

The scheme for obtaining the coatings is shown in Figure 1.

**Figure 2:**
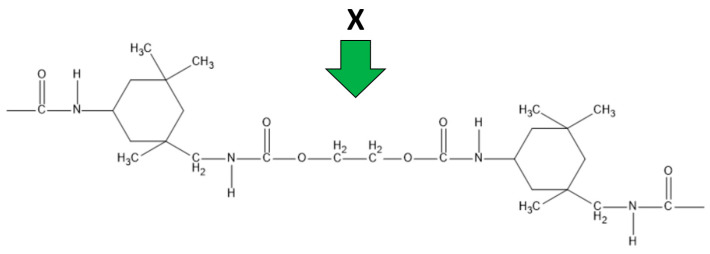
Structural formula of the crosslinking agent.

Figure 2 shows the structure of the cross-linking agent responsible for cross-linking the magnetic hydrogel particles.

Substrates for coating synthesis were specimens made of ABS plastic (BestFilament, Moscow, Russia) printed on a QIDI Q1 Pro FDM 3D printer (QIDI, Ruian, China). The printing parameters were as follows: 100% infill, a build plate temperature of 110 °C, a nozzle temperature of 250 °C, and a chamber temperature of 50 °C. Prior to printing, the ABS filament was dried in an oven at 70 °C for 10 h to remove moisture. Two types of specimens were printed: dog-bone specimens (for measuring mechanical properties according to the ISO 527-1:2019 standard [34]) and discs with a diameter of 20 mm and a thickness of 2 mm (for measuring thermal conductivity). ABS plastic was selected due to the high sensitivity of its mechanical properties to the negative effects of ultraviolet radiation, making it a convenient model for practical evaluation of coating efficiency [35,36].

**Figure 3:**
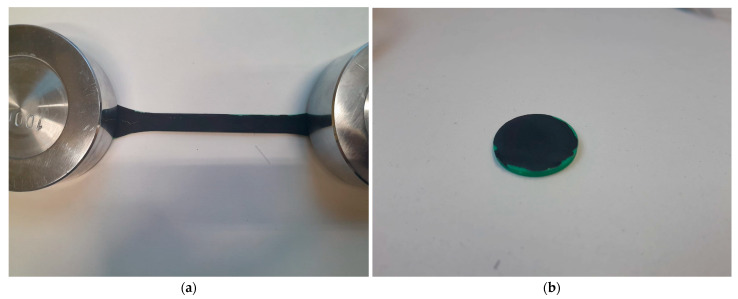
Appearance of the coatings applied to the ABS plastic substrates. (**a**) Dog-bone specimen; (**b**) Sample for thermal conductivity measurement.

The appearance of the synthesized samples is shown in Figure 3.

### 2.3. Characterization Methods of Initial Few-Layer Graphene and Coatings Based on FLG

Electronic images of FLG, as well as coatings based on FLG, were obtained by scanning electron microscopy (SEM) using a Tescan Mira 3-M instrument (Tescan, Brno-Kohoutovice, Czech Republic). The accelerating voltage was 20 kV. To obtain images of the initial FLG, the powder particles were deposited onto carbon tape. Images of the FLG at high magnification were obtained using a transmission electron microscope (TEM) FEI Tecnai G2 30 S-TWIN (FEI, Hillsboro, OR, USA). The accelerating voltage was set to 50 kV. For TEM studies of the initial FLG, a suspension was prepared based on isopropyl alcohol (0.05 wt.%) by treating it in an ultrasonic bath (22 kHz) for 5 min. The resulting suspension was then deposited onto a carbon-coated grid and dried at 50 °C for one hour.

The particle size distribution of FLG was measured by laser diffraction using a Mastersizer 2000 instrument (Malvern, Westborough, MA, USA). The operating principle of the method is based on the diffraction of a laser beam by the measured particles as it passes through the suspension [37]. A plate-like particle model was used for the measurement. To determine the particle size of the FLG, an aqueous suspension (0.05 wt.%) was prepared by treatment in an ultrasonic bath (22 kHz) for 5 min.

The IR spectra of FLG and coatings based on FLG were obtained using an Infralume FT-08 spectrometer (Lumex Marketing, Saint Petersburg, Russia) by the attenuated total reflectance (ATR) method on a PIKE attachment (Pike Technologies, Fitchburg, WI, USA). To obtain the FTIR spectrum of the carbon material, the carbon powder was mixed with water to form a thick paste. Then, 1 µL of the paste was applied to the ATR crystal at a temperature of 45 °C. The spectra were recorded until water evaporation was complete.

Raman spectra of FLG and coatings based on FLG were obtained with an “Ntegra Spectra” spectrometer (“NT-MDT”, Moscow, Russia). The spectrometer was equipped with a 532 nm diode laser, a 100× objective, and a grating with 600 grooves/mm. The measurements were carried out at room temperature. To prevent sample heating, a beam with a power density of <50 W/cm^2^ was used. Spectra were recorded at several random points; each graph represents the average spectra from the sample.

The thermal conductivity of the coatings was studied using the monotonic cooling method with a KITT “composite” instrument (“Teplofon” company, Krasnoyarsk, Russia). The measurements were conducted at 25 °C.

Sample exposure to ultraviolet light was carried out in a SONACME SB-UV-A chamber (SONACME, Dongguan, China, wavelength 340 nm, total power 60 W) for 96 h.

The primary criteria for assessing the effect of ultraviolet radiation were the change in tensile strength and elongation at break (before and after UV treatment). Tensile strength tests were performed on a universal tensile testing machine HSL-UT-50PC (Dongguan Hongjin Test Instrument Co., Dongguan, China). The loading rate was 10 mm/min.

## 3. Results

**Figure 4:**
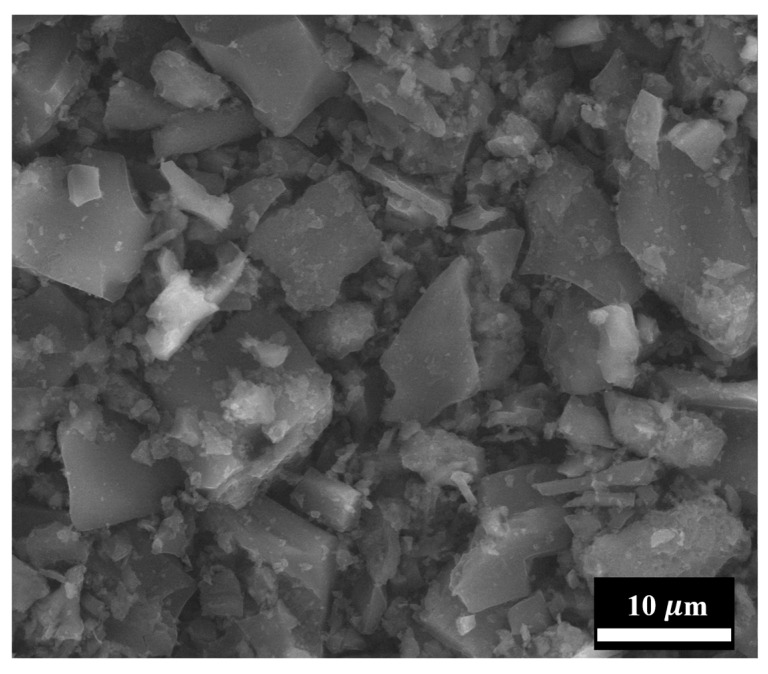
SEM image of the FLG powder particles. The scale bar is 10 µm.

**Figure 5:**
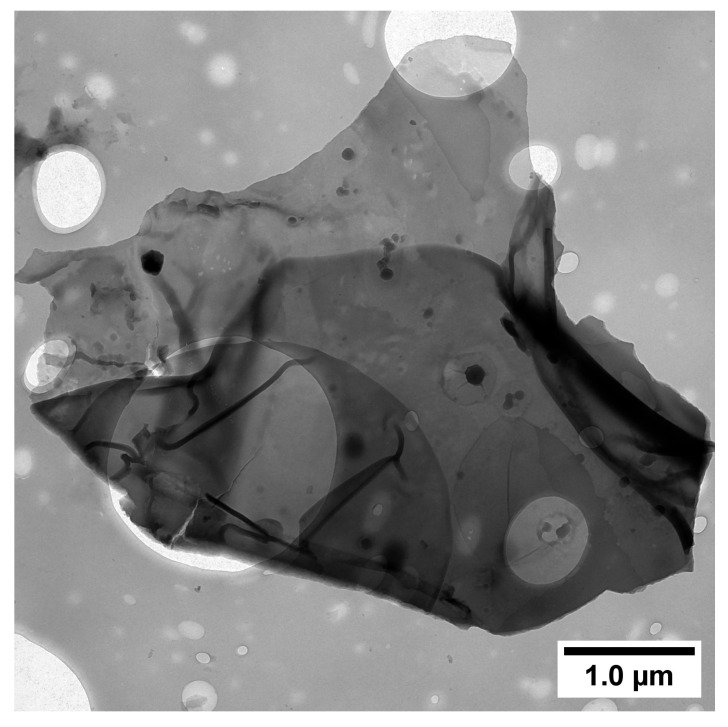
TEM image of the FLG particle. The scale bar is 1 µm.

Figure 4 and Figure 5 show electron microscopy images of the initial FLG particles obtained by scanning electron microscopy and transmission electron microscopy, respectively.

**Figure 6:**
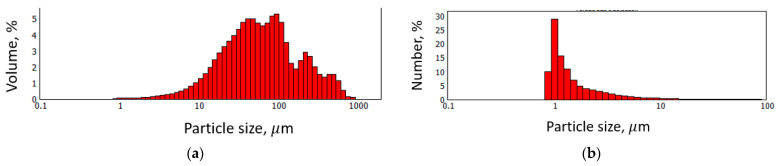
Particle size distribution of FLG. (**a**) Volume-based particle size distribution; (**b**) Number-based particle size distribution.

As can be seen from Figure 4, the FLG powder particles consist of large aggregates (several tens of microns), which is typical for GNS in powder form. However, as shown in Figure 5, the FLG particles themselves are several microns in size. Furthermore, Figure 5 reveals that due to their small thickness, the carbon-coated grid is clearly visible behind the FLG particle, which is consistent with the structure of FLG. Laser diffraction studies were conducted to determine the precise linear particle sizes, and the results are presented in Figure 6.

As can be seen from Figure 6, although particles with linear sizes up to hundreds of microns are present in the sample (Figure 6a), the proportion of such particles is small, and most particles have a size of about 1 µm (Figure 6b). The obtained data are in good agreement with the results from transmission electron microscopy (TEM).

**Figure 7:**
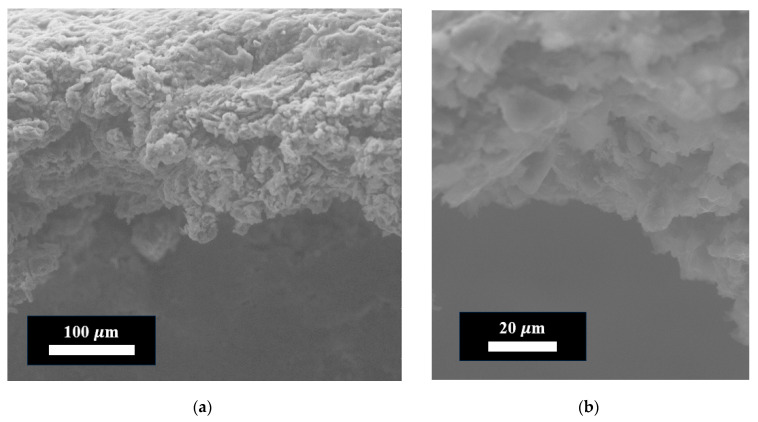
Electron images of the coating obtained by the chemical cross-linking method of FLG particles. (**a**) Linear scale = 100 µm; (**b**) Linear scale = 20 µm.

Figure 7 shows an electron image of the synthesized coating obtained by the chemical cross-linking method of FLG particles.

As can be seen from Figure 7, the coating thickness is 250 ± 10 µm. It can also be concluded that the orientation of particles in the synthesized coating is random. Furthermore, it can be noted that the degree of FLG particle aggregation changed. However, it was not possible to quantify this change in particle size using laser diffraction due to the requirement for sample preparation as a suspension, which would inherently alter the agglomeration state.

**Figure 8:**
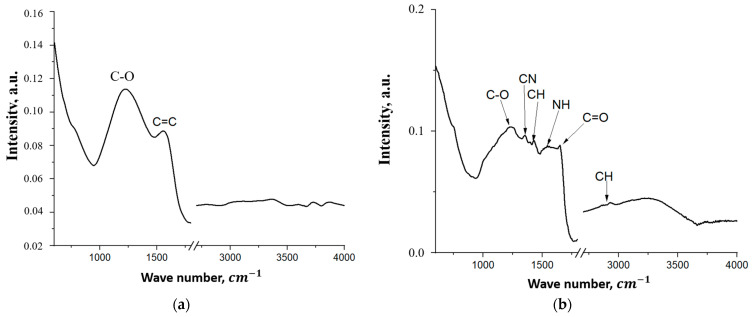
FT-IR spectrum of the initial FLG (**a**) coating obtained by the chemical cross-linking method of FLG particles (**b**).

Figure 8 shows the FT-IR spectra of the FLG particles and the coating based on them.

The FT-IR spectrum of the few-layer graphene (Figure 8a) shows two broad bands—the band at 1224 cm^−1^ is attributed to C-O vibrations, and the band at 1558 cm^−1^ is attributed to C=C bond vibrations [38]. The significant breadth of these bands is likely due to a wide range of alcohol and ether groups formed as a result of uncontrolled processes during the SHS.

The FT-IR spectrum of the coating based on few-layer graphene (Figure 8b) shows bands corresponding to the following bond vibrations: C-O-C at 1237 cm^−1^, C-N in the urethane group at 1350 cm^−1^, C-H at 1430 cm^−1^, 2865 cm^−1^, and 2931 cm^−1^, and N-H and C=O in the urethane group at 1530 cm^−1^ and 1650 cm^−1^, respectively [39]. The appearance of these bonds is a result of the chemical cross-linking of the FLG particles. The N-H and C=O bonds are weakly expressed, likely due to overlap with the broad C=C band from the few-layer graphene.

**Figure 9:**
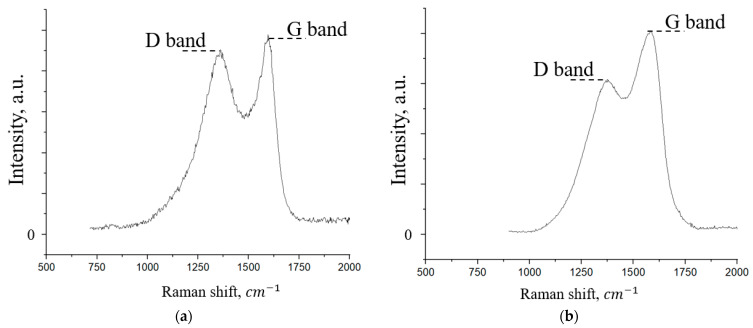
Raman spectrum of the initial FLG (**a**) and coating obtained by the chemical cross-linking method of FLG particles (**b**).

Figure 9 shows the Raman spectra of the initial FLG powder and the synthesized coating.

As can be seen from Figure 9, the spectra show the D peak and G peak, which are typical for GNS [40]. The appearance of the G peak is due to the in-plane stretching vibrations of carbon atoms in the graphene lattice. In contrast, the appearance of the D peak is due to the presence of various defects in the structure of the graphene nanosheets. Comparing their intensities allows for a semi-quantitative assessment of the defect density in the graphene nanosheets [41]. The intensity ratio of the D and G peaks (I_D_/I_G_) for the FLG particles in powder form is 0.95, while for the FLG particles in the coating, this ratio is 0.76, indicating a reduction in the defect density of the material [42]. One type of defect that contributes to the D peak is the edges of graphene planes, whose chemical bonds are terminated by various groups (-OH, -COOH, etc.). Considering that the coatings are formed by chemical cross-linking via these terminal surface groups on the graphene planes, it can be hypothesized that this process leads to a reduction in defect density.

Table 1 presents the results of the thermal conductivity measurements for the coating.

As can be seen from Table 1, the thermal conductivity of the substrate in the longitudinal direction has a value typical for ABS plastic, 0.17 W/(m × K) [43]. In the transverse direction, however, the thermal conductivity of the substrate is significantly lower, which is associated with the layer-by-layer printing of the sample and thermal losses at the interlayer boundaries. The thermal conductivity of the sample in the longitudinal direction increased significantly (from 0.17 to 244 W/(m × K)), which is because the thermal conductivity became determined by the thermal conductivity of the coating. Given that the thermal conductivity of the coating is orders of magnitude higher than that of the substrate, the potential contribution of the substrate’s thermal conductivity to the final measurements can be neglected.

Table 2 presents the results of UV resistance measurements for samples made of pure ABS and those with a coating made of FLG particles.

As can be seen from Table 2, the mechanical properties of the ABS plastic specimens are comparable with data from other studies on FDM-printed ABS materials [44]. Exposure to UV radiation can lead to either a decrease or an increase in the mechanical parameters of a polymer, depending on the predominant degradation mechanism. In both cases, the change in mechanical properties results from destructive processes within the polymer matrix [45]. Therefore, the most objective evidence of the coating’s protective effectiveness is the stability of the polymer material’s mechanical parameters after UV exposure. As expected, UV treatment of the pristine ABS plastic led to a significant deterioration of its mechanical characteristics due to polymer chain degradation [46,47]. In contrast, the mechanical properties of the ABS specimens with a protective coating remained unchanged. This indicates an absence of polymer chain degradation and, consequently, demonstrates the coating’s high barrier properties against UV radiation.

Let us consider the possible reasons for such properties of the coatings. It should be noted that the obtained thermal conductivity of the coating made from few-layer graphene particles in the longitudinal direction, while comparable to the thermal conductivity of metals (for example, the thermal conductivity of aluminum is 237 W/(m × K)), is not record-breaking for this type of coating. For instance, in the work [48], the authors reported a coating based on reduced graphene oxide with a thermal conductivity of 2850 W/(m × K) in the parallel direction. As noted in review [49], the final thermal conductivity of a coating based on graphene nanosheets is determined by three main factors: particle orientation, particle defect density, and the interaction between particles. Based on the electron microscopy data (Figure 3) and the results of Raman studies (Figure 5), it can be assumed that while the FLG particles are strongly bonded to each other, they have a significant number of defects and are not aligned in any specific direction, which prevents the full realization of the coating’s thermophysical potential. However, as shown in the review [50], graphene nanosheets exhibit strong UV absorption and are also capable of reflecting it due to their layered structure. Therefore, the imperfections of the synthesized coatings described above do not significantly affect the efficiency of UV protection, unlike their effect on thermal conductivity.

**Figure 10:**
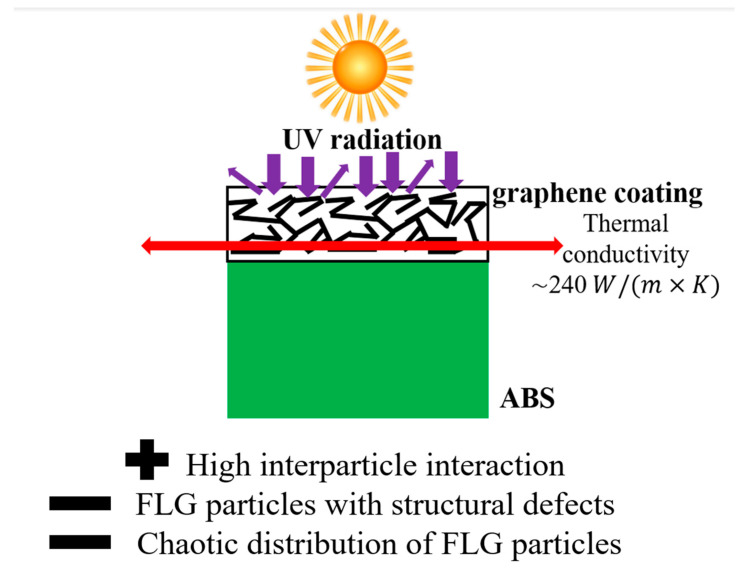
Structural model of the coating actually synthesized in this work from few-layer graphene particles via chemical cross-linking.

Figure 10 presents a model of the structure of the coating actually synthesized in this work, also made from graphene particles.

## 4. Conclusions

This study successfully demonstrates the feasibility of fabricating protective coatings from few-layer graphene (FLG) produced cost-effectively from lignin via Self-propagating High-temperature Synthesis (SHS). The developed coatings, based on chemically cross-linked FLG particles, exhibit a high thermal conductivity of 244 W/(m·K) and provide effective protection against UV degradation, highlighting their potential for practical applications.

To fully realize this potential, key challenges must be addressed. The current coatings are limited by the defect density of the FLG particles and their tendency to form aggregates during the coating process, which disrupts a more ordered structure. Therefore, future work will focus on two primary strategies: reducing the defect density in the initial FLG particles through high-temperature annealing and optimizing the coating process, including a reduction in thickness, to achieve a more uniform and ordered particle arrangement.

## Figures and Tables

**Table 1:** Results of thermal conductivity measurements of the samples.

Through-Plane Thermal Conductivity of ABS Substrate, W/(m × K)	In-Plane Thermal Conductivity of ABS Substrate, W/(m × K)	In-Plane Thermal Conductivity of FLG Coating, W/(m × K)
0.10 ± 0.02	0.17 ± 0.02	244 ± 8

**Table 2:** Results of the strength properties measurements of the samples.

	Initial ABS	Initial ABS After UV Exposure	ABS + FLG Coating	ABS + FLG Coating After UV Exposure
Tensile strength, MPa	29 ± 1	19 ± 1	29 ± 1	30 ± 1
Elongation at break, %	7 ± 1	2 ± 1	7 ± 1	7 ± 1

## Data Availability

The original contributions presented in the study are included in the article. Further inquiries can be directed to the corresponding author.

## Abbreviations

The following abbreviations are used in this manuscript:

FLG	few-layer graphene
GNS	graphene nanostructures
CVD	Chemical vapor deposition
SHS	self-propagating high-temperature synthesis
